# New York College of Dentistry

**Published:** 1866-09

**Authors:** 


### NEW YORK COLLEGE OF DENTISTRY.

The first annual announcement of this institution is on our table, and also in the advertising pages of this Number.

If the plan of the organization is fully carried out, it will certainly be very efficient in the accomplishment of good for the profession.

The experience of others has doubtless been of some advantage in the organization of this school; for while they are small in the beginning, this professes to *start* at almost full growth. There is no objection to the size or strength of a new being, provided all the parts are of good structure and healthy; it is a very common impression, however, that exercise has something to do with permanent growth and strength.

We hail the establishment of this College as an epoch in the profession, and especially that part of it in New York and vicinity We have been of the opinion that an institution of this kind should have been established in that city long ago.

We hope and believe that a career of great usefulness and prosperity awaits this enterprise.

